# The Effect of Degradation of Soda Lignin Using Pd/SO_4_^2−^/ZrO_2_ as a Catalyst: Improved Reactivity and Antioxidant Activity

**DOI:** 10.3390/polym11071218

**Published:** 2019-07-21

**Authors:** Shengming Zhang, Guizhen Fang, Haitao Chen, Qian Lang

**Affiliations:** 1 College of Engineering, Northeast Agricultural University, Harbin 150030, China; 2 Agricultural Engineering Postdoctoral Research Station, Northeast Agricultural University, Harbin 150030, China; 3 College of Material Science and Engineering, Northeast Forestry University, Harbin 150040, China

**Keywords:** soda lignin, Pd/SO_4_^2−^/ZrO_2_ catalyst, degradation, antioxidant activity, reactivity

## Abstract

To the value-added application of the soda lignin by improving its reactivity and antioxidant activity, a self-made Pd/SO_4_^2^^−^/ZrO_2_ catalyst was used to catalyze the degradation reaction of soda lignin. The catalyst was loaded with the palladium of 1.47 wt.% while retaining the super acidity of SO_4_^2^^−^/ZrO_2_. The reaction condition was determined as follows: the dioxane-water solution was selected as the reaction solution, the addition amount of the catalyst was 5 wt.% of the soda lignin, the system was heated at 100 °C for 4 h under a hydrogen pressure of 3 MPa. The reactivity of the catalyzed-soda lignin compared to the soda lignin before the reaction was significantly improved: the values of phenolic hydroxyl groups and total hydroxyl groups were increased by 35.3% and 97.1%, respectively, and the value of methoxy groups was decreased by 13%. Approximately 63.3% of the β-O-4 bonds were cleaved, which resulted in a reduction of the weight average molecular weight from 8200 g·mol^−1^ to 4900 g·mol^−1^. At the same time, the EC_50_ values of the catalyzed-soda lignin on DPPH (1,1-diphenyl-2-picrylhydrazyl) and ABTS^+^ (2,2′-azino-bis(3-ethylbenzothiazoline-6-sulfonate) radicals scavenging were decreased by 20.6% and 32.6%, respectively, and the reducing power of catalyzed-soda lignin at the absorption value of 0.5 was increased by 10.5%. The Pd/SO_4_^2^^−^/ZrO_2_ catalyst works by breaking the β-O-4 linkages and degrading the methoxy groups. The catalyzed-soda lignin exhibits the possibility of being used as the antioxidants, grafting precursors, adhesive additives, and raw materials for lignin/polymer composites.

## 1. Introduction

Soda lignin is a type of technical lignin derived from the black liquor. Due to the high degree of polymerization and low reactivity, the soda lignin is difficult to be utilized. As an aromatic polymer, soda lignin consists of three structural units, such as the guaiacyl (G), syringyl (S), and p-hydroxyphenyl (H) units, and it also contains some active functional groups. After the appropriate processing, soda lignin may hopefully be used as a raw material for synthetic fine chemicals or lignin-based polymers. Currently, some researchers have succeeded in degrading the lignin into alkylbenzenes, alkylphenols, and other phenolic molecules [1,2,3]. However, the reaction conditions are generally harsh and energy-consuming. In comparison, improving the reactivity of lignin is another effective method for its value-added utilization [4]. In previous studies, we developed some acid catalysts and hydrocracking catalysts to degrade the β-O-4 bond of lignin under relatively mild conditions. The reactivity of the catalyzed-lignin was improved in the reaction, and we also found that the antioxidant activity of catalyzed-lignin was improved.

The precious metal catalyst palladium (Pd) is a hydrocracking catalyst with good efficiency and selectivity [5,6]. To et al. [7] reported that the C-C sigma bond could be converted to the C-H bond by hydrogenolysis with the Pd/C catalyst. Zhu et al. [8] used the Pd/C catalyst to degrade the model compounds of no benzyl lignin, and they found that the β-O-4 bonds could be rapidly cleaved. In previous studies, we used the self-made Pd/C catalyst to activate and degrade the soda lignin by hydrogenolysis and obtained good effects: the value of phenol hydroxyl groups increased by 28–56.6%, the number average molecular weight (*M*_n_) significantly reduced from 3600 g·mol^−1^ to 2300g·mol^−1^, and the DPPH free radical scavenging rate was improved [9,10]. In the catalytic system, the Pd is an active component, and the activated carbon acts only as a carrier and has no catalytic action.

Furthermore, the SO_4_^2−^/ZrO_2_ is an environment-friendly solid acid [11]. It is useful for the lignin degradation, and the Bronsted acid sites of the SO_4_^2−^/ZrO_2_ catalyst provide the active center [12,13]. After the reaction, the value of phenolic hydroxyl groups of the soda lignin can be increased by 15.3%, resulting in an improvement in antioxidant activity. Based on the SO_4_^2−^/ZrO_2_ catalyst, we prepared a dual-function catalyst, named CuO/SO_4_^2−^/ZrO_2_, by dipping and roasting method, which was more effective than the SO_4_^2−^/ZrO_2_ catalyst for lignin degradation reaction [14]. Besides the increase in the values of phenolic hydroxyl groups and total hydroxyl groups, the value of methoxy groups of the soda lignin decreased by 10.2%, and the S/G unit ratio reduced from 1.5 to 1.2 during the reaction. In theory, the Pd catalyst is a more effective hydrocracking catalyst with the higher catalytic activity than the CuO catalyst. Therefore, we considered using SO_4_^2−^/ZrO_2_ as a carrier and catalyst, using Pd component instead of CuO component, and constructing the Pd/SO_4_^2−^/ZrO_2_ catalyst, to further improve the reactivity and antioxidant activity of the soda lignin.

In this study, the Pd/SO_4_^2−^/ZrO_2_ catalyst was prepared in two steps: the SO_4_^2−^/ZrO_2_ carrier was prepared by the dipping and roasting method, and the Pd was loaded onto the SO_4_^2−^/ZrO_2_ carrier using a formaldehyde reduction method. The soda lignin, extracted from the black liquor dry matter, was catalyzed through the hydrogenolysis routes. The effect of reaction conditions was first studied in the dioxane-water solution using the values of phenolic hydroxyl groups and total hydroxyl groups as the index. After that, the performance of the catalyst was evaluated in three other reaction solutions, such as the sodium hydroxide (NaOH) solution, 1-butyl-3-methylimidazolium chloride ([Bmim]Cl) solution, and dimethyl sulphoxide (DMSO). The structure of lignins was characterized by using the analytical methods, such as the spectral analysis technology, nuclear magnetic technique, and chemical analysis. The antioxidant activity of lignins was evaluated by using methods, such as the ABTS^+^ (2,2′-azino-bis(3-ethylbenzothiazoline-6-sulfonate) radical scavenging, DPPH (1,1-diphenyl-2-picrylhydrazyl) radical scavenging, and reducing power. Finally, the mechanism of lignin degradation was discussed.

## 2. Materials and Methods

### 2.1. Materials

The soda lignin was extracted from the dry matter of black liquor (the source wasthe wheat straw; purchased from Shandong Tralin Paper Co., Ltd., Quanlin, China) as described by Zhang et al. [14]. The 1 kg dry matter was dissolved in the 10 L distilled water. The insoluble impurities in the mixture were removed by decompression filtration; the pH value of the filtrate was adjusted to 5 by adding 2 mol·L^−1^ hydrochloric acid solution; the mixture was decompression filtered, and the lignin filter cake was washed to neutral by adding the distilled water while pumping; the lignin cake was dried at 45 °C for 48 h under the vacuum to obtain the soda lignin. The yield of the soda lignin was 12.5 wt.% of the dry matter with a purity of 93.3% (in the soda lignin, the content of acid-insoluble lignin, acid-soluble lignin, total sugars, and ash were 89.7%, 3.6%, 1.8%, and 4.9%, respectively). The zirconyl chloride octahydrate (ZrOCl_2_·8H_2_O, 98%) was purchased from Sigma-Aldrich Co. LLC, Shanghai, China. The palladium chloride (PdCl_2_, Pd content of 59%), 1, 4-dioxane (99.5%), NaOH (96%), and DMSO (99%) were purchased from Sinopharm Chemical Reagent Co., Ltd., Shanghai, China. The [BMIM]Cl (99%) was produced by Shanghai Chengjie Chemical Co., Ltd., Shanghai, China.

### 2.2. Catalysts Preparation

The solid acid carrier SO_4_^2−^/ZrO_2_ was prepared using the dipping and roasting method, which can be found elsewhere [13]. The Pd component was loaded onto the SO_4_^2−^/ZrO_2_ carrier using a formaldehyde reduction method. Firstly, the 0.15 g PdCl_2_ was dissolved in the 20 mL hydrochloric acid solution (3.2 mol·L^−1^) at 80 °C; the 3 g SO_4_^2−^/ZrO_2_ was added to the 40 mL distilled water at 90 °C. Next, the palladium chloride solution was added dropwise to the SO_4_^2−^/ZrO_2_ mixture with rapid stirring. After the temperature of the mixture was cooled to 20 °C, the pH value of the mixture was adjusted to 9–10 by adding the NaOH solution (3.2 mol·L^−1^). Then, the mixture was allowed to stand for 1 h, the formaldehyde was added dropwise to the mixture, and the mixture was aged for half an hour. Finally, the mixture was decompression filtered. The filter cake was washed to neutral with the distilled water at 50 °C. After drying at 110 °C for 4 h, the Pd/SO_4_^2−^/ZrO_2_ catalyst was obtained.

### 2.3. Degradation Reaction

In the experiments using the dioxane-water solution (1, 4-dioxane: distilled water, 4:1, v/v) as the reaction solution, the 4 g soda lignin was dissolved in the solution, and the 0.2 g Pd/SO_4_^2−^/ZrO_2_ catalyst (5 wt.% of the soda lignin) was added to the mixture. The mixture was transferred into a polytetrafluoroethylene liner and placed into a 1L–reactor kettle (Dalian-Controlled Plant Co., Ltd., Dalian, China). The reaction system was filled with the initial 3 MPa hydrogen pressure and then was heated. The temperature was investigated at 60 °C, 80 °C, 90 °C, 100 °C, 110 °C, and 120 °C, respectively; the effect of the time was studied at 2 h, 4 h, and 6 h, respectively; the catalyst addition was investigated at 0, 2.5 wt% (0.1 g), 5 wt% (0.2 g), 7.5 wt% (0.3 g), and 10 wt% (0.4 g). When the reaction was completed, the catalyst was separated by decompression filtration. The filtrate of the products was concentrated by rotary evaporator and transferred to a surface dish. After drying at 45 °C for 24 h under the vacuum, the catalyzed-soda lignin was obtained.

In the experiments using the NaOH solution (0.05 mol·L^−1^) and DMSO solution as the reaction solution, respectively, the products were collected by using the acid precipitation method. In brief, after the catalyst was separated, the filtrate was adjusted to pH 5 and then decompression filtered; the filter cake was washed to neutral by adding the distilled water while pumping. In the experiments using the [BMIM]Cl solution as the reaction solution, the products were collected by rotary evaporation. The 1 L distilled water was added into the filtrate at 50 °C. After decompression filtration, the filter cake of the products was dried under the vacuum to obtain the catalyzed lignin.

### 2.4. Catalysts Characterization

For the SO_4_^2−^/ZrO_2_ carrier, the acid strength was evaluated by the Hammett indicator method, which can be found elsewhere [12]. The acid strength of the Pd/SO_4_^2−^/ZrO_2_ was evaluated by the NH_3_-TPD (ammonia temperature-programmed desorption) method. The 0.1 g catalyst was pretreated in the helium stream at 500 °C for 2 h. After the temperature dropped to 100 °C, the sample was purged with the ammonia for 0.5 h. The purge gas was switched to the helium gas for 2 h to remove the physically adsorbed ammonia. The temperature was raised to 800 °C at the heating rate of 15 °C per minute, and the helium flow rate was 40 mL per minute. The XRD (X-ray diffraction) patterns were recorded in a range of 5°–90 ° on a Rigaku-D/MAX-2200VPC (Rigaku Co., Tokyo, Japan). The scanning speed was 6° per minute. The content of palladium of the Pd/SO_4_^2−^/ZrO_2_ catalyst was measured using a Persee TAS-990 atomic absorption spectrumapparatus (Beijing Purkinje General Instrument Co., Ltd., Beijing, China). The pore structure of the catalysts was measured by the BET (Brunauer-Emmett-Teller measurement) method on an ASAP 2020-Physisorption Analyzer (Micromeritic Instruments Co., Norcross, GA, USA). The morphology of the catalysts was recorded on an FEI QUATA200 scanning electron microscopy equipment (FEI Co., Eindoven, The Netherlands).

### 2.5. Products Characterization

The total hydroxyl groupsvalue of the lignin samples was determined by the method of chemical titration, which can be found elsewhere [14]. The lignin samples were dissolved in the acetylation reagent and acetylated for 24 h, and then titrated with the NaOH solution to calculate the total hydroxyl groups value. The phenolic hydroxyl groups value of the lignin samples was determined by the method of ultraviolet spectrophotometry, which can be found in our previous work [15]. The molecular weight of the products was measured by GPC (gel permeation chromatography) method on an Agilent 1100 HPLC apparatus (Agilent Technologies Inc., Palo Alto, CA, USA). The test method was as follows: the 5 mg acetylated lignin sample was dissolved in the 4 mL tetrahydrofuran; the 79911GP–101 column and 79911GP–104 column were connected in series; the flow rate of the tetrahydrofuran eluent was 1 mL·min^−1^; the column temperature was 30 °C; the injection volume was 50 μL. A Persee FTIR-650 spectrometer (Tianjin Gangdong Sci. & Tech. Development Co., Tianjin, China) was used to capture the FT-IR spectra of the products. The test method was as follows: the number of scans was 16 times; the resolution was 2 cm^−1^; the scanning range was 4000–500 cm^−1^. The 2D ^1^H-^13^C HSQC NMR spectra and ^1^H NMR spectra of the products were recorded on a Bruker-AVIII-400MHz spectrometer (Bruker Co., Fällanden, Switzerland). The detailed test parameters can be found in our previous work [14].

### 2.6. Antioxidant Activity Evaluation

The test method of the DPPH radical scavenging rate can be found in the references [13]. Briefly, the 1 mL lignin solution and 4 mL DPPH solution were mixed at 20 °C. The mixture was allowed to stand in the dark. After 30 min, the UV absorbance value was recorded at 517 nm to calculate the scavenging rate. The assay method of the ABTS^+^ radical scavenging rate can be found in the references [13]. The different concentration of lignin solution was mixed with the ABTS phosphate buffer solution. After 10 min, the UV absorbance value was recorded at 734 nm to calculate the scavenging rate. For the reducing power method, the 2.5 mL sodium phosphate buffer (pH 6.6), 1 mL lignin solution, and 2.5 mL potassium ferricyanide solution (1% by weight) were mixed at 50 °C. After 20 min, the mixture and 2.5 mL trichloroacetic acid (10% by volume) were mixed. After centrifugation, the 2.5 mL supernatant, 0.5 mL ferric chloride solution (0.1%by weight), and 2.5 mL distilled water were mixed. The UV absorbance value of the mixture was recorded at 700 nm as the reducing power [16].

## 3. Results

### 3.1. Characterization of the Catalysts

**Figure 1:**
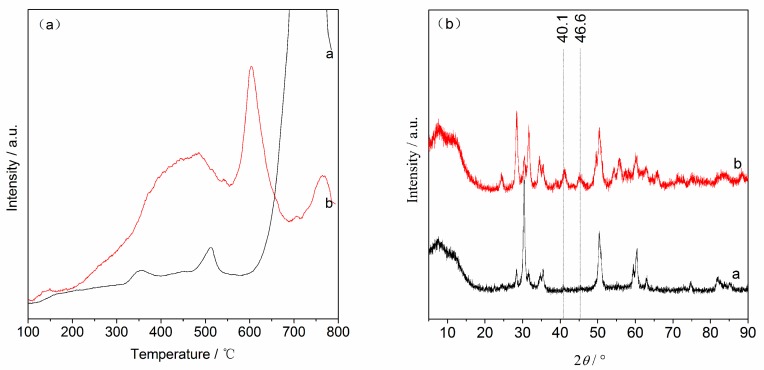
Temperature-programmed desorption) patterns (**a**) and XRD patterns (**b**) of the SO_4_^2−^/ZrO_2_ (a—black peak) and Pd/SO_4_^2−^/ZrO_2_ (b—red peak).

The acid strength H_0_ = −14.06 of the SO_4_^2−^/ZrO_2_ solid acid carrier was measured by the Hammett indicator method. The detailed measurement process can be found in our previous work [12]. Since the Hammett indication method was not suitable for evaluating the acid strength of dark solid acids, such as the gray-black Pd/SO_4_^2−^/ZrO_2_, the NH_3_-TPD method was chosen to be used. The NH_3_-TPD patterns of SO_4_^2−^/ZrO_2_ and Pd/SO_4_^2−^/ZrO_2_ catalysts are shown in Figure 1. The temperature of the NH_3_ desorption peak is related to the acid center intensity of the samples. The strong acid center usually leads to a high-temperature desorption peak [17,18]. From Figure 1, the three desorption peaks of SO_4_^2−^/ZrO_2_ catalyst at 170 °C, 350 °C, and 530 °C were attributed to the weak, medium-strong, and strong acid sites, respectively. For the Pd/SO_4_^2−^/ZrO_2_ catalyst, the weak acid sites appeared at 140 °C, and the medium-strong and strong acid sites appeared at 450 °C and 600 °C, respectively. It indicates that the Pd/SO_4_^2−^/ZrO_2_ catalyst maintains the acid strength of SO_4_^2−^/ZrO_2_ solid acid.

**Figure 2:**
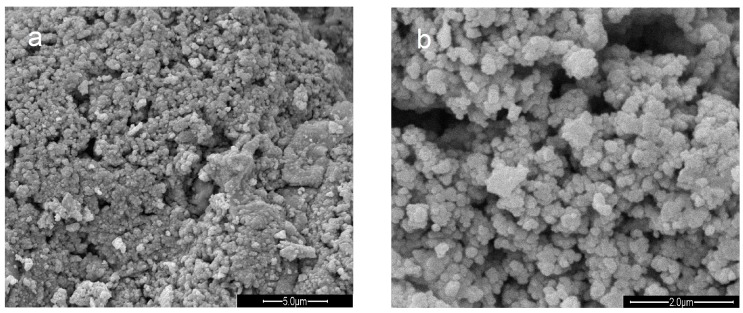
Scanning electron microscopy of the Pd/SO_4_^2−^/ZrO_2_ catalyst ((**a**) magnification 5k; (**b**) magnification 20k).

The XRD patterns of the Pd/SO_4_^2−^/ZrO_2_ and SO_4_^2−^/ZrO_2_ are shown in Figure 1. Compared with the SO_4_^2−^/ZrO_2_ pattern, two peaks newly appearing at 40.1° and 46.6° in the Pd/SO_4_^2−^/ZrO_2_ pattern were assigned to the Pd (111) and Pd (200) planes [19,20]. The XRD results indicated that the Pd was successfully loaded onto the SO_4_^2−^/ZrO_2_ carrier. The BET results showed that the Pd/SO_4_^2−^/ZrO_2_ catalyst had a BET surface area of 23.60 m^2^·g^−1^ and a microporous surface area of 0.87 m^2^·g^−1^. The atomic absorption analysis showed the loading content of the Pd was 1.42 wt.%in the catalyst, which was 47.33% of the theoretical loading content. The SEM image of the Pd/SO_4_^2−^/ZrO_2_ catalyst is shown in Figure 2. From Figure 2, the spherical Pd metal particles were uniformly supported on the solid acid carrier.

### 3.2. Characterization of the Products

#### 3.2.1. Reaction Conditions

**Figure 3:**
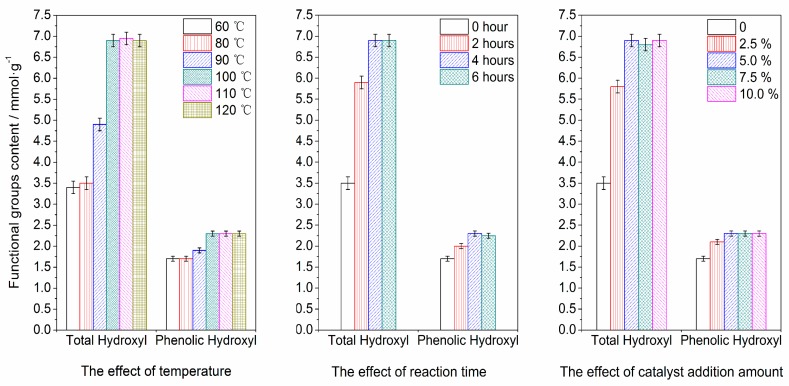
The effects of reaction conditions on the degradation reaction.

In the reaction using dioxane-water as a solution, the effects of temperature, reaction time, and catalyst addition amount on the reaction are shown in Figure 3 (The detailed values are shown in Table S1). The initial hydrogen pressure for all reactions was 3 MPa. The effect of temperature was investigated under the following conditions: the catalyst addition amount was 5 wt.% of the soda lignin, and the system was heated for 4 h. The maximum valueof phenolic hydroxyl groups of 2.30 mmol·g^−1^ was obtained at the reaction temperature of 100 °C. Meanwhile, the value of the total hydroxyl groups attained 6.90 mmol·g^−1^. In the case of eliminating measurement errors, the values of the phenolic hydroxyl groups and total hydroxyl groups showed no increment with the increase in temperature. Therefore, the temperature was chosen to be 100 °C.

The effect of reaction time was studied under the condition as follows: the catalyst addition amount was 5 wt.% of the soda lignin, and the system was heated at 100 °C. From 0 to 4 h, the value of phenolic hydroxyl groups increased with the reaction time, and the changing trend of total hydroxyl value was the same as that of phenolic hydroxyl groups value. At the reaction time of 4 h, the maximum values of phenolic hydroxyl groups and total hydroxyl groups reached 6.90 mmol·g^−1^ and 2.30 mmol·g^−1^, respectively.

After the temperature and reaction time were determined, the effect of the amount of catalyst added on the reaction was measured. When the catalyst addition amount reached 5 wt.% of the soda lignin, the maximum values of functional groups, total hydroxyl groups of 6.90 mmol·g^−1^, and phenolic hydroxyl groups of 2.30 mmol·g^−1^ were obtained. Therefore, the optimal catalyst addition amount was chosen to be 5 wt.%.

Besides, the effect of Pd/SO_4_^2−^/ZrO_2_ (Pd/SZ) catalyst on the degradation reaction was further investigated in three other solutions. The effects of solutions on the reaction are shown in Table 1. The results showed that the Pd/SO_4_^2−^/ZrO_2_ catalyst had the catalytic activity in all solutions. In the experiment using dioxane-water as a solution, the phenolic hydroxyl groups value of the catalyzed-soda lignin reached a maximum of 2.30 mmol·g^−1^. The maximum value of 7 mmol·g^−1^ of the total hydroxyl groups was obtained in the experiment using DMSO as a solution. The weight percentage of catalyzed-soda lignin to soda lignin was defined as the product yield. Compared to the sample L14, sample L16, and sample L18, the sample L4 had the highest product yield of 82.33 wt.%. For the experiments using DMSO solution and NaOH solution, respectively, the product was collected by the acid precipitation method. In the step of the products collecting, the filter cake of acid-insoluble lignin was collected, but the acid-soluble lignin dissolved in the filtrate was lost, which resulted in a loss of the product of about 2.5–4% by weight. For the experiment using [BMIM]Cl solution, a few of the lignin products were dissolved in the ionic liquid and were difficult to be separated, resulting in a loss of the product of about 3% by weight. The sample (L4) with the lowest weight average molecular weight (*M*_w_) of 4900 g·mol^−1^ was obtained in the reaction using dioxane-water as a solution.

#### 3.2.2. FT-IR Spectra

**Figure 4:**
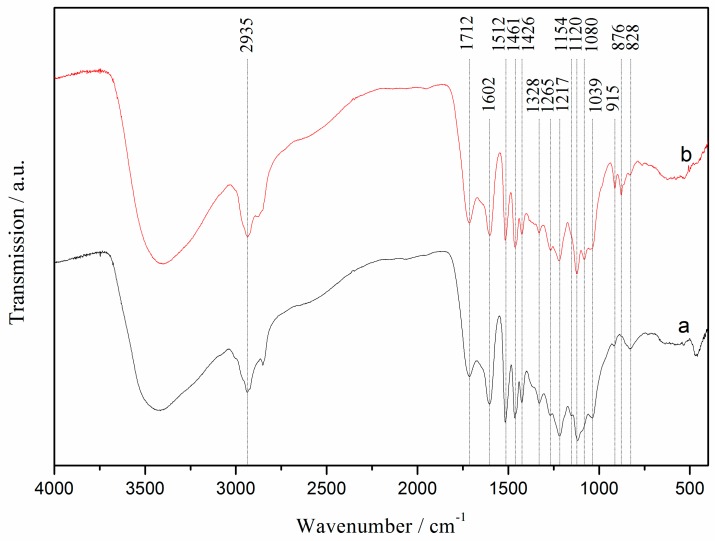
FT-IR spectra of the soda lignin (a; L0) and catalyzed-soda lignin (b; L4).

The FT-IR spectra of the lignin samples before (L0) and after the reaction (L4) are shown in Figure 4. The attribution of infrared peaks was determined by the description of Faxi [21] and Jahan et al. [22]. The bonds at 1602 cm^−1^, 1512 cm^−1^, and 1426 cm^−1^ were the characteristic peaks of the lignin phenyl-propane skeleton. The sample L4 showed the peaks at 1039 cm^−1^ (C-H in-plane deformation from the guaiacyl units), 828 cm^−1^ (C-H out-of-plane deformation from the syringyl units), 2935 cm^−1^ (C-H stretching from the methane, methylene, or methyl groups), and 1461 cm^−1^ (theasymmetric bending deformation of the methyl and methylene groups). Compared with the sample L0, the intensity of the above peaks did not change significantly, which indicated that the phenylpropane structure of the sample L4 did not change during the reaction. In both spectra, the C-O deformation was observed at 1080 cm^−1^ and 1120 cm^−1^. The C=O stretching and C-C plus C-O stretch were observed at 1712 cm^−1^ and 1217 cm^−1^, respectively. The intensity of the above peaks did not change substantially. The peak at 1154 cm^−1^ was assigned to the conjugated C=O in ester groups. After the reaction, the intensity of the peak at 1154 cm^−1^ was significantly weakened. This phenomenon is because the ester groups of lignin sample were converted to the hydroxyl groups in the reaction. In spectra (b), the intensity of the peak at 915 cm^−1^ (C-H stretching out of aromatic ring plane) was stronger than that of spectra (a). The new peak at 876 cm^−1^ was observed in spectra (b), which might attribute to the C-H stretching out of the plane. Compared with the intensity of the peaks in spectra (a), the peak at 1328 cm^−1^ (the C-O stretching in the syringyl units) in spectra (b) was significantly weaker, while the peak at 1265 cm^−1^ (the C-O stretching in theguaiacyl units) was stronger. This phenomenon might be because the S/G ratio of the catalyzed-soda lignin was reduced during the reaction.

#### 3.2.3. NMR Spectroscopy

**Figure 5:**
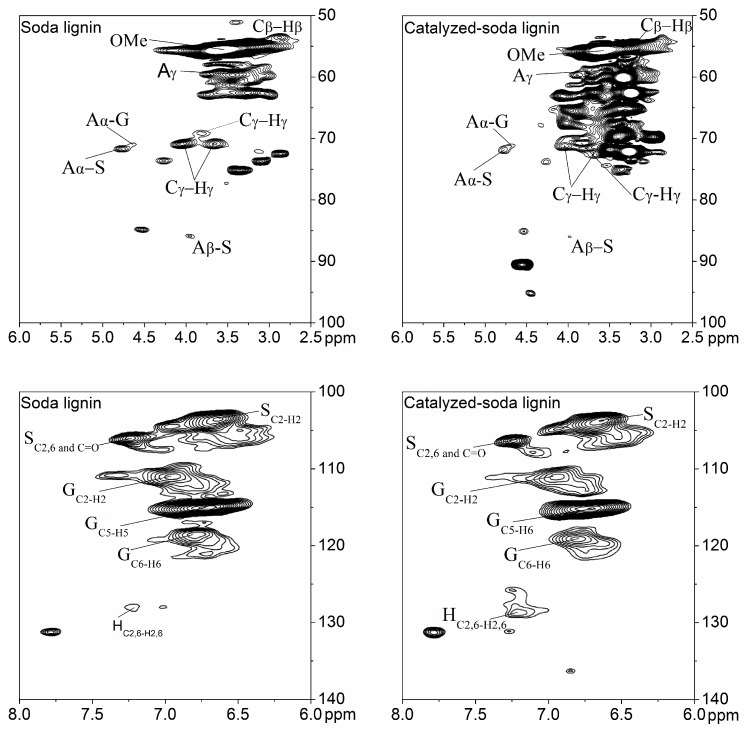
2D-HSQC NMR spectra of the soda lignin and catalyzed-soda lignin.

The 2D-HSQC spectra of the samples L0 and L4 are depicted in Figure 5. The major structural units and chemical bonds attribution of the lignin samples were determined according to the references [14,23,24]. The signals of the S units were observed at δ_C_/δ_H_ 104.3/6.73 ppm (C_2_-H_2_) and δ_C_/δ_H_ 106.6/7.30 ppm (C_2, 6_ and C=O). The signals of the G units were observed at δ_C_/δ_H_ 119.3/6.80 ppm(C_6_-H_6_), 115.2/6.98 ppm (C_5_-H_5_), 115.2/6.74 ppm(C_5_-H_5_), and 111.3/7.03 ppm (C_2_-H_2_). The signals of H units were observed at δ_C_/δ_H_ 127.7/7.17 ppm (C_2,6_-H_2,6_). The signals of δ_C_/δ_H_ 59.8/3.49 ppm (A_γ_), 72.3/4.90 ppm (A_α-S_), 71.5/4.77 ppm (A_α-G_), and δ_C_/δ_H_ 86.4/4.15 ppm (A_β-S_) were assigned to the β-O-4 linkage. The signals of δ_C_/δ_H_ 71.4/3.85 ppm (C_γ_-H_γ_), 54/3.10 ppm (C_β_-H_β_), and 71.4/4.22 ppm (C_γ_-H_γ_) were assigned to the β-β linkage. The relative amounts of the linkages and structural unit ratio were calculated by the Topspin NMR software, which can be found elsewhere [25]. For the sample L4, the content of β-O-4 linkages was 3.13/100Ar, which was 63.3% less than that of the sample L0 of 8.53/100Ar. Meantime, the content of β-β linkages of the sample L4 was 7/100Ar, which was 17.8% more than that of the sample L0 of 5.94/100Ar. Moreover, the S/G unit ratio of the soda lignin decreased from 1.67 to 1.12 during the reaction. Besides that, the value of OCH_3_ groups was determined based on the ^1^H-NMR spectra, as described by Mousavioun et al. [26]. The ^1^H-NMR spectra of the sample L0 and sample L4 are depicted in Figure S1. The results showed that the content of the methoxyl groups of the sample L0 was 6 mmol·g^−1^, which was 13% less than that of the sample L0 of 6.9 mmol·g^−1^.

### 3.3. Antioxidant Activity

**Figure 6:**
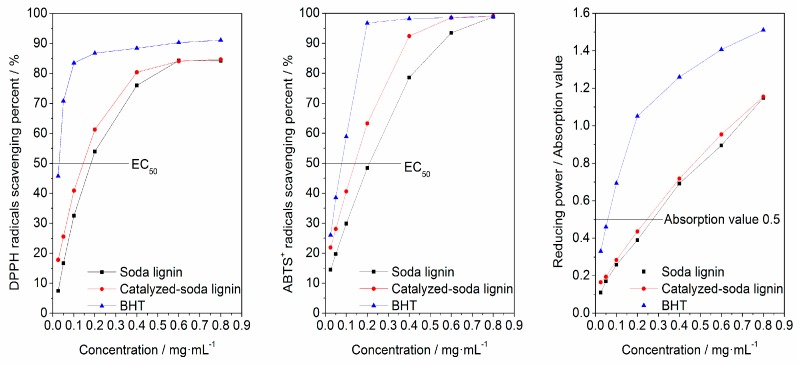
DPPH radicals scavenging and ABTS^+^ radicals scavenging percent and the reducing power of lignin samples. BHT: butylated hydroxytoluene.

The antioxidant activity of the lignin samples before (L0) and after the reaction (L4) are shown in Figure 6. A common antioxidant BHT (butylated hydroxytoluene) was chosen to be the reference. For the same concentration of the lignin samples, the DPPH and ABTS radical scavenging rates of the sample L4 were stronger than that of the sample L0 in the concentration range of 0.025–0.6 mg·mL^−1^. When the concentration reached 0.6 mg·mL^−1^, the DPPH free radical scavenging rate of two lignin samples reached the maximum value of 84%, which was lower than that of the BHT (91.1%). For the ABTS method, the maximum scavenging rate of 99% of two lignin samples and BHT was obtained at the concentration of 0.8 mg·mL^−1^. When the concentration range was 0.025–0.8 mg·mL^−1^, the reduction power of the sample L4 was stronger than that of the sample L0 but lower than that of the BHT. The EC_50_ value is the concentration of the sample at the free radical scavenging rate of 50%. The sample with a stronger antioxidant capacity typically exhibits the lower EC_50_ value [27]. In the DPPH radical scavenging rate test, the EC_50_ value of 145.2 μg·mL^−1^ of the sample L4 was 20.6% lower than that of the sample L0 of 182.8 μg·mL^−1^. In the ABTS^+^ radical scavenging rate test, the EC_50_ value of 95.1 μg·mL^−1^ of the sample L4 was 32.6% lower than that of the sample L0 of 141.1 μg·mL^−1^. The strength of the reducing power was determined by comparing the concentration of the lignin samples at the UV absorbance value of 0.5. The value of 246.7 μg·mL^−1^ of the sample L4 at the UV absorbance value of 0.5 was 10.4% lower than that of the sample L0 of 275.3 μg·mL^−1^.

## 4. Discussion

The total hydroxyl value and phenolic hydroxyl value can reflect the reactivity of lignin samples. Pinheiro et al. [28] and Zhang et al. [29] prepared the phenol-formaldehyde resin and bio-phenol-hydroxymethylfurfural resins by using the optimized acetosolv lignins and phenolated de-polymerized hydrolysis lignin, respectively. It has been shown that the lignin, which has the higher phenolic hydroxyl groups value, the higher total hydroxyl groups value, the lower value of methoxyl groups, and the lower molecular weight, is more suitable for producing the phenolic resins. Su et al. [30] used the trimethyl lignin quaternary amine salt (TLQA) and carboxymethylated polyvinyl alcohol (CMPVA) to prepare a lignin-polyvinyl alcohol film through layer-by-layer self-assembly. The TLQA containing 3.56% nitrogen by weight was obtained by the reaction of alkaline lignin and quaternary ammonium monomer. The phenolic hydroxyl group is the reactive sites of lignin. It means that the TLQA containing the higher nitrogen can be obtained by using the lignin raw materials with higher phenolic hydroxyl content. Zhang et al. [31] used the sodium alginate and soda lignin to synthesize a lignin quaternary ammonium salts that could be used as flocculants. Similar to Su’s research, the phenolic hydroxyl group is the reactive sites in the synthesis of lignin quaternary ammonium salts (QL). So, it is possible to obtain a higher yield of QL by using the lignin, which contains the more phenolic hydroxyl groups.

In this study, the sample L4 had the highest value of the phenolic hydroxyl groups, the highest product yield, and the lowest *M*_w_. The total hydroxyl groups value of sample L4 was 6.90 mmol·g^−1^, which was very close to the 7 mmol·g^−1^ of sample L18.The allowable error range for measuring the total hydroxyl value is ±0.15 mmol·g^−1^.Based on above-mentioned findings, the optimal reaction condition was determined as follows: the dioxane-water solution was selected as the reaction solution, the addition amount of the catalyst was 5 wt.% of the soda lignin, the system was heated at 100 °C for 4h under a hydrogen pressure of 3 MPa. Compared with the other lignin samples, the sample L4 showed the possibility to be further developed and utilized, such as the grafting precursor of lignin quaternary ammonium salts, the additive for adhesives, and the raw material of lignin/polymer composites.

Besides, the Pd/SO_4_^2−^/ZrO_2_ catalytic system showed the best effect in improving the antioxidant activity of soda lignin compared to the catalytic system (CuO/SO_4_^2−^/ZrO_2_ catalytic system [14] and [BMIM]Cl catalytic system [32]) we used previously. The EC_50_ value of the Pd/SO_4_^2−^/ZrO_2_ catalyzed-soda lignin (sample L4) on DPPH radicals scavenging was decreased by 20.6%, which was 19.8% higher than that of the [BMIM]Cl catalyzed-soda lignin (17.2%). The EC_50_ value of the Pd/SO_4_^2−^/ZrO_2_ catalyzed-soda lignin (sample L4) on ABTS^+^ radicals scavenging was decreased by 32.6%, which was 129.6% and 7.2% higher than that of the [BMIM]Cl catalyzed-soda lignin (14.2%) and CuO/SO_4_^2−^/ZrO_2_ catalyzed-soda lignin (30.4%), respectively. The reducing power of Pd/SO_4_^2−^/ZrO_2_ catalyzed-soda lignin at the absorption value of 0.5 was increased by 10.5%, which was 16.7% stronger than that of the CuO/SO_4_^2−^/ZrO_2_ catalyzed-soda lignin (9%). Therefore, the Pd/SO_4_^2−^/ZrO_2_ catalyzed-soda lignin might be used as an antioxidant in the cosmetics and polymeric materials. The Pd/SO_4_^2−^/ZrO_2_catalyst is very easy to recycle. Future work could consider a further analysis of the reuse of the catalyst.

Compared with three other solutions, the [BMIM]Cl solution had a certain ability to degrade the soda lignin. Research has been shown that the β-O-4 bonds of lignin can be hydrolyzed under the acid conditions, and the new structure named lignin-bound Hibbert ketones can be formed [33,34]. In previous studies, it was proven that the soda lignin might undergo the acidic hydrolysis in the [BMIM]Cl, which led to the degradation of soda lignin and an increase in total hydroxyl groups value [32]. However, the effect of Pd/SO_4_^2−^/ZrO_2_ catalyst on the degradation reaction of soda lignin was significantly stronger than that of the [BMIM]Cl.

**Scheme 1:**
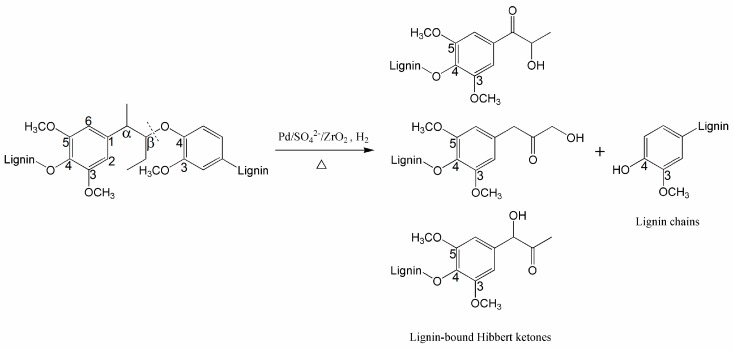
Reaction mechanism of the β-O-4 linkages cleavage.

In previous studies, we proved that the content of β-O-4 bonds of the SO_4_^2−^/ZrO_2_-catalyzed lignin was reduced by 25% after the reaction, resulting in a reduction of the *M*_w_ from 7700 g·mol^−1^ to 5800 g·mol^−1^ [14]. In this study, the content of β-O-4 bonds of the Pd/SO_4_^2−^/ZrO_2_-catalyzed lignin was reduced by 63.3%, and the *M*_w_ wasdecreased from 8200 g·mol^−1^ to 4900 g·mol^−1^. Therefore, the effect of the Pd/SO_4_^2−^/ZrO_2_ catalyst on lignin degradation was significantly stronger than that of the SO_4_^2−^/ZrO_2_, and the addition of the Pd component resulted in the further degradation of the β-O-4 bonds. After the β-O-4 bonds break, the formed structures of lignin-bound Hibbert ketones and lignin chains led to an increase in the total hydroxyl groups and phenolic hydroxyl groups. The degradation mechanism is shown in Scheme 1.

**Scheme 2:**
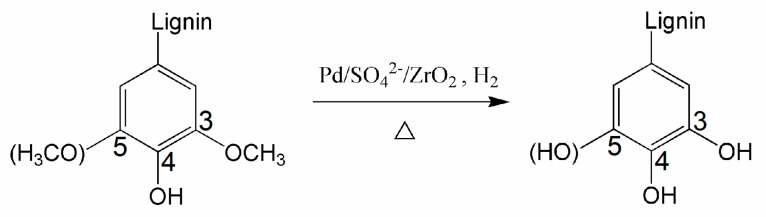
Possible reaction mechanism of the methoxy groups degradation.

In the experiment using SO_4_^2−^/ZrO_2_ as a catalyst, the S/G unit ratio of the catalyzed-soda lignin did not change compared with that of the soda lignin before the reaction. However, in the experiment using Pd/SO_4_^2−^/ZrO_2_ as a catalyst, the S/G unit ratio of catalyzed-soda lignin decreased from 1.67 to 1.12, and the value of the methoxy groups decreased by 13%. The results showed that a small portion of the methoxy groups on the soda lignin benzene ring was degraded during the reaction using Pd/SO_4_^2−^/ZrO_2_ as a catalyst. Generally, when a one-component metal catalyst, such as thePd catalyst and CuO catalyst, is used for lignin hydrogenolysis, the effective catalytic temperature usually needs to reach 300 °C, and the lignin can be degraded into the small moleculecompounds [35,36]. But there are also reports that the multi-component catalysts can achieve lignin degradation under relatively mild conditions. For example, Zhang et al. [37] reported that the β-O-4 type C-O chemical bonds of the organosolv lignin were cleaved by NiPd bimetallic catalystsat 130 °C and the phenolic monomers were found in the products. In this study, the soda lignin was degraded under the combined catalysis of Pd and SO_4_^2−^/ZrO_2_. The newly formed phenolic hydroxyl groups might appear at the C_3_ and C_5_ positions on the benzene ring. The possible degradation mechanism is shown in Scheme 2.

## 5. Conclusions

A bifunctional catalyst Pd/SO_4_^2−^/ZrO_2_ was successfully prepared in this study. The catalyst was loaded with the palladium of 1.47 wt.% while retaining the super acidity of SO_4_^2−^/ZrO_2_. The reaction condition was determined as follows: the dioxane-water solution was selected as the reaction solution, the addition amount of the catalyst was 5 wt.% of the soda lignin, the system was heated at 100 °C for 4 h under a hydrogen pressure of 3 MPa. Approximately 63.3% of the β-O-4 bonds were cleaved, which resulted in a reduction of the lignin’s weight average molecular weight from 8200 g·mol^−1^ to 4900 g·mol^−1^. The formed structures of lignin-bound Hibbert ketones and lignin chains led to an increase in the total hydroxyl groups and phenolic hydroxyl groups by 97.1% and35.3%, respectively. Compared with the soda lignin before the reaction, the S/G unit ratio of the catalyzed-soda lignin decreased from 1.67 to 1.12, and the methoxy groups content reduced by 13%, which might be due to the degradation of the methoxy groups on lignin’s benzene ring. The EC_50_ values of the catalyzed-soda lignin on DPPH and ABTS^+^ radicals scavenging were decreased by 20.6% and 32.6%, respectively, and the reducing power of catalyzed-soda lignin at the absorption value of 0.5 was increased by 10.5%. The catalyzed-soda lignin showed the possibility to be further developed and utilized, such as the antioxidants, grafting precursors, adhesive additives, and raw materials for lignin/polymer composites.

## Figures and Tables

**Table 1:** The effects of solutions on the reaction.

Sample	Catalyst	Solution	Functional Groups Value (mmol·g^−1^)		*M*_w_ (g·mol^−1^)	*M*_n_ (g·mol^−1^)	*M*_w_/*M*_n_	Yield (wt.%)
			Total Hydroxyl (±0.15)	Phenol Hydroxyl (±0.06)				
L0	—	—	3.50	1.70	8200	3700	2.22	—
L9	None	Dioxane-water	3.50	1.70	8200	3700	2.22	99.99
L4	Pd/SZ	Dioxane-water	6.90	2.30	4900	2400	2.04	82.33
L13	None	NaOH	3.50	1.70	8000	3600	2.22	97.50
L14	Pd/SZ	NaOH	6.80	1.90	5200	2400	2.17	79.24
L15	None	[BMIM]Cl	5.30	1.90	6900	3000	2.30	80.00
L16	Pd/SZ	[BMIM]Cl	6.90	2.20	5100	2300	2.22	81.03
L17	None	DMSO	3.50	1.70	8400	3700	2.27	96.00
L18	Pd/SZ	DMSO	7.00	2.20	5400	2700	2.00	74.33

## Supplementary Materials

The following are available online at https://www.mdpi.com/2073-4360/11/7/1218/s1, Figure S1: The 1H-NMR spectra of lignin samples before (a, L0) and after (b, L4) the reaction, Table S1: The effects of reaction conditions on the degradation reaction.
