# Supplementary material for: The Effect of Degradation of Soda Lignin Using Pd/SO42−/ZrO2 as a Catalyst: Improved Reactivity and Antioxidant Activity
Source: Polymers (Basel). 2019 Jul 21;11(7):1218. doi: 10.3390/polym11071218 (PMC6681089; doi:10.3390/polym11071218)
Supplement: Supplementary file 1 [file polymers-11-01218-s001.pdf]

polymers

MDPI

Table S1. The effects of reaction conditions on the degradation reaction.

Sample	Temperature (°C)	Reaction time (hour)	Catalyst addition amount (wt.%)	Functional groups value (mmol·g⁻¹) Total hydroxyl (±0.15)	Functional groups value (mmol·g⁻¹) Phenol hydroxyl (±0.06)
L0	—	—	—	3.50	1.70
L1	60	4	5	3.40	1.70
L2	80	4	5	3.50	1.70
L3	90	4	5	4.90	1.90
L4	100	4	5	6.90	2.30
L5	110	4	5	6.95	2.30
L6	120	4	5	6.90	2.30
L7	100	2	5	5.90	2.00
L8	100	6	5	6.90	2.25
L9	100	4	0	3.50	1.70
L10	100	4	2.5	5.80	2.10
L11	100	4	7.5	6.80	2.30
L12	100	4	10	6.90	2.30

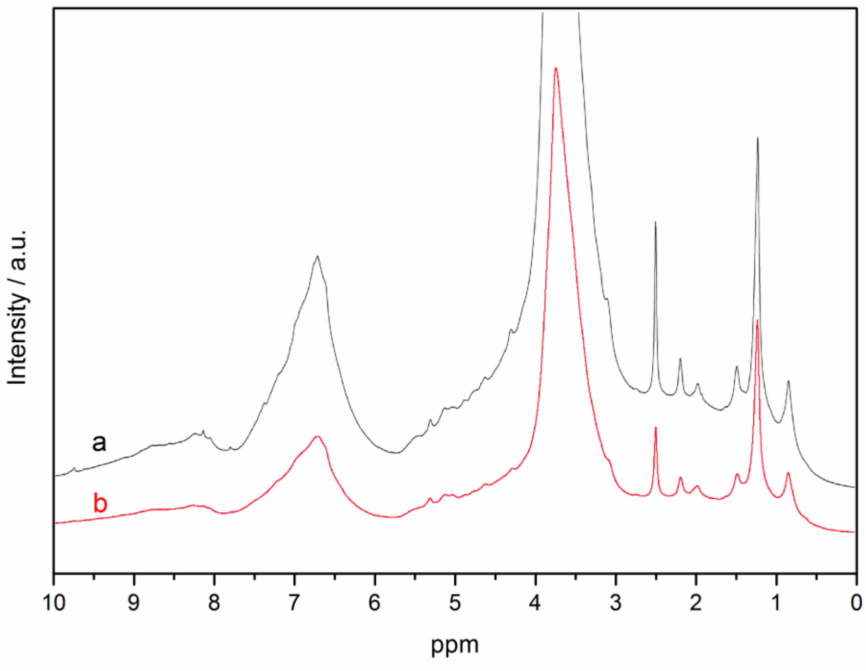

Figure S1. The ^{1}H-NMR spectra of lignin samples before (a, L0) and after(b, L4)the reaction.
